# Facing privacy in neuroimaging: removing facial features degrades performance of image analysis methods

**DOI:** 10.1007/s00330-019-06459-3

**Published:** 2019-11-05

**Authors:** A. de Sitter, M. Visser, I. Brouwer, K. S. Cover, R. A. van Schijndel, R. S. Eijgelaar, D. M. J. Müller, S. Ropele, L. Kappos, Á. Rovira, M. Filippi, C. Enzinger, J. Frederiksen, O. Ciccarelli, C. R. G. Guttmann, M. P. Wattjes, M. G. Witte, P. C. de Witt Hamer, F. Barkhof, H. Vrenken, on behalf of the MAGNIMS Study Group and Alzheimer’s Disease Neuroimaging Initiative

**Affiliations:** 1 Department of Radiology and Nuclear Medicine, Amsterdam Neuroscience Amsterdam UMC, location VUmc, Amsterdam, the Netherlands; 2 Department of Radiotherapy, The Netherlands Cancer Institute, Amsterdam, the Netherlands; 3 Department of Neurosurgery, Amsterdam UMC, location VUmc, Amsterdam, the Netherlands; 4 Department of Neurology, Medical University of Graz, Graz, Austria; 5 Department of Neurology, University Hospital, Kantonsspital, Basel, Switzerland; 6 Unitat de Ressonància Magnètica (Servei de Radiologia), Hospital universitari Vall d’Hebron, Universitat Autònoma de Barcelona, Barcelona, Spain; 7 Neuroimaging Research Unit, Institute of Experimental Neurology, Division of Neuroscience, San Raffaele Scientific Institute, UniSR, Milan, Italy; 8 Division of Neuroradiology, Vascular and Interventional Radiology, Department of Radiology, Medical University of Graz, Graz, Austria; 9 Department of Neurology, Glostrup University Hospital, Copenhagen, Denmark; 10 UK/NIHR UCL-UCLH Biomedical Research Centre, Institute of Neurology, UCL, London, UK; 11 Center for Neurological Imaging, Department of Radiology, Brigham and Women’s Hospital, Harvard Medical School, Boston, MA USA; 12 Department of Diagnostic and Interventional Neuroradiology, Hannover Medical School, Hannover, Germany; 13 Institutes of Neurology & Healthcare Engineering, UCL, London, UK

**Keywords:** Magnetic resonance imaging, Ethics, Database, Neuroimaging, Privacy

## Abstract

**Background:**

Recent studies have created awareness that facial features can be reconstructed from high-resolution MRI. Therefore, data sharing in neuroimaging requires special attention to protect participants’ privacy. Facial features removal (FFR) could alleviate these concerns. We assessed the impact of three FFR methods on subsequent automated image analysis to obtain clinically relevant outcome measurements in three clinical groups.

**Methods:**

FFR was performed using QuickShear, FaceMasking, and Defacing. In 110 subjects of Alzheimer’s Disease Neuroimaging Initiative, normalized brain volumes (NBV) were measured by SIENAX. In 70 multiple sclerosis patients of the MAGNIMS Study Group, lesion volumes (WMLV) were measured by lesion prediction algorithm in lesion segmentation toolbox. In 84 glioblastoma patients of the PICTURE Study Group, tumor volumes (GBV) were measured by BraTumIA. Failed analyses on FFR-processed images were recorded. Only cases in which all image analyses completed successfully were analyzed. Differences between outcomes obtained from FFR-processed and full images were assessed, by quantifying the intra-class correlation coefficient (ICC) for absolute agreement and by testing for systematic differences using paired *t* tests.

**Results:**

Automated analysis methods failed in 0–19% of cases in FFR-processed images versus 0–2% of cases in full images. ICC for absolute agreement ranged from 0.312 (GBV after FaceMasking) to 0.998 (WMLV after Defacing). FaceMasking yielded higher NBV (*p* = 0.003) and WMLV (*p* ≤ 0.001). GBV was lower after QuickShear and Defacing (both *p* < 0.001).

**Conclusions:**

All three outcome measures were affected differently by FFR, including failure of analysis methods and both “random” variation and systematic differences. Further study is warranted to ensure high-quality neuroimaging research while protecting participants’ privacy.

**Key Points:**

• *Protecting participants’ privacy when sharing MRI data is important*.

• *Impact of three facial features removal methods on subsequent analysis was assessed in three clinical groups*.

• *Removing facial features degrades performance of image analysis methods*.

**Electronic supplementary material:**

The online version of this article (10.1007/s00330-019-06459-3) contains supplementary material, which is available to authorized users.

## Introduction

Sharing participant image data can offer many benefits to neuroradiological research: a better understanding of diseases can be achieved by access to larger participant populations in combined multicenter datasets; researchers without access to their own data on a specific disease can still contribute to its understanding by using shared datasets; and methodological improvements can be stimulated by publicly shared benchmark datasets.

However, for shared data, it is crucial to protect participants’ privacy. Image files should not contain identifying information such as name, date of birth, or any national or hospital-based registration numbers. Such data are often saved in metadata or even filenames of magnetic resonance (MR) images and should be removed before sharing. Unfortunately, this is not enough to alleviate privacy concerns, since typical structural magnetic resonance imaging (MRI) provides good enough skin to air contrast and spatial resolution to perform facial recognition from a 3D-rendered version of the image, whether by the human eye or using facial recognition software [1–5]. Therefore, in addition to identifying metadata, it has been suggested that facial features should also be removed, and this has been widely embraced [6–9]. However, it is not yet clear whether the removal of the facial features affects subsequent measurement of quantitative indices of brain pathology.

Therefore, the current study assessed the impact of facial features removal (FFR) on clinically relevant outcome measurements. We selected three FFR methods that are publicly available, well documented, and have been used in data sharing initiatives [10, 11]: QuickShear [12], FaceMasking [13], and Defacing [14]. We assessed their effects on clinically relevant outcome measures in three different diseases: normalized brain volumes (NBV) in Alzheimer’s disease (AD), white matter lesion volumes (WMLV) in multiple sclerosis (MS), and tumor volumes (GBV) in glioblastoma patients.

## Materials and methods

### Subject

Subjects in this study were obtained from three different dataset: for AD, a dataset from the ADNI study (http://adni.loni.usc.edu/) [15]; for MS, a multicenter dataset from the MAGNIMS Study Group (https://www.magnims.eu/) [16]; and for treatment-naïve glioblastoma patients, a clinical dataset from the PICTURE project collected in the Amsterdam UMC, location VUmc, in Amsterdam, the Netherlands. Primary studies were approved by the respective local ethics committee for all three datasets. A summary of the demographics is given in Supplementary Table 1.

#### Alzheimer’s disease

Data used in the preparation of this article were obtained from the ADNI database. The ADNI was launched in 2003 as a public-private partnership, led by Principal Investigator Michael W. Weiner, MD. The primary goal of ADNI has been to test whether serial MRI, positron emission tomography, other biological markers, and clinical and neuropsychological assessment can be combined to measure the progression of mild cognitive impairment (MCI) and early Alzheimer’s disease (AD).

From the ADNI1 dataset, we selected the subset of subjects who had a 3-Tesla (T) magnetization-prepared rapid acquisition gradient echo (MPRAGE) baseline MRI, which is a subset of the 562 subjects that are in the ADNI1 dataset [15, 17]. This subset included in total 110 (23% female) subjects with an average age of 75 (range 60–87) years. This dataset included 39 healthy elderly controls, 52 patients with mild cognitive impairment, and 19 patients with AD.

#### Multiple sclerosis

For MS, a multicenter dataset of the MAGNIMS Study Group was previously used to study iron accumulation in deep gray matter [18] and lesion segmentation software performance [16]. The dataset consisted of 70 patients (67% female), scanned in six different MAGNIMS centers. On average, the age was 34.9 (range 17–52) years. The mean disease duration from onset was 7.6 (range 1–28) years and the disease severity was measured using the Expanded Disability Status Scale (EDSS) on the day of scanning; patients had a median EDSS score of 2 (range 0.0–6.5) [19].

#### Glioblastoma

For glioblastoma, a total of 84 (38% female) patients were selected from a cohort treated at the Neurosurgical Center of the Amsterdam UMC, location VUmc, Amsterdam, the Netherlands, in 2012 and 2013. On average, the age was 61.4 (range 21–84) years. All patients had histopathologically confirmed WHO grade IV glioblastoma. The preoperative MRI was made on average within 1 week before resection.

### MRI procedure

In the MS and AD datasets, all imaging was performed on 3-T whole-body MR systems, and for imaging of the glioblastoma dataset on 1.5- and 3-T MR systems. The protocol for the AD dataset included a 3D T1-weighted sequence, while the protocol for MS included a 3D T1-weighted sequence, as well as a 2D fluid-attenuated inversion recovery (FLAIR) sequence. The protocol for glioblastoma contained a 3D T1-weighted post contrast–enhanced scan, 3D FLAIR, and 2D T2-weighted and non-enhanced 2D T1-weighted sequence. In Table 1, more details are listed on data acquisition of the datasets.

**Table 1:** Details on the data acquisition of the AD, MS, and glioblastoma datasets

					Sequence parameters			
Dataset	Scanner brands	Scanner types	Field strength (Tesla)	Sequence	TR (ms)	TE (ms)	TI (ms)	Slice thickness (mm)
AD	Siemens GE Medical Systems Philips	Not known	3	3D T1	2300–3000	2.98	853–900	1.2
MS	Siemens Philips	Trio Achieva	3	2D FLAIR 3D T1	8000–11,000 6.9–2300	69–136 2.8–298	2400–2800 815–900	3.0 1.0
Glioblastoma	Siemens GE Medical Systems Toshiba Philips	Sonata or Avanto Signa HDxt or DISCOVERY MR750 Titan3T Panorama HFO or Achieva	1.5 and 3	2D FLAIR 3D T1* 2D T1 2D T2	6500 2300–2700 520–600 5190–8670	355 4.5–5.0 8.0–12.0 93–101	2200 950	1.3 1.0–1.5 5 5

*Post contrast (0.2 mmol/kg)

*AD*, Alzheimer’s disease; *MS*, multiple sclerosis; *FLAIR*, fluid-attenuated inversion recovery; *TR*, repetition time; *TE*, echo time; *TI*, inversion time

### Facial features removal methods

**Fig. 1:**
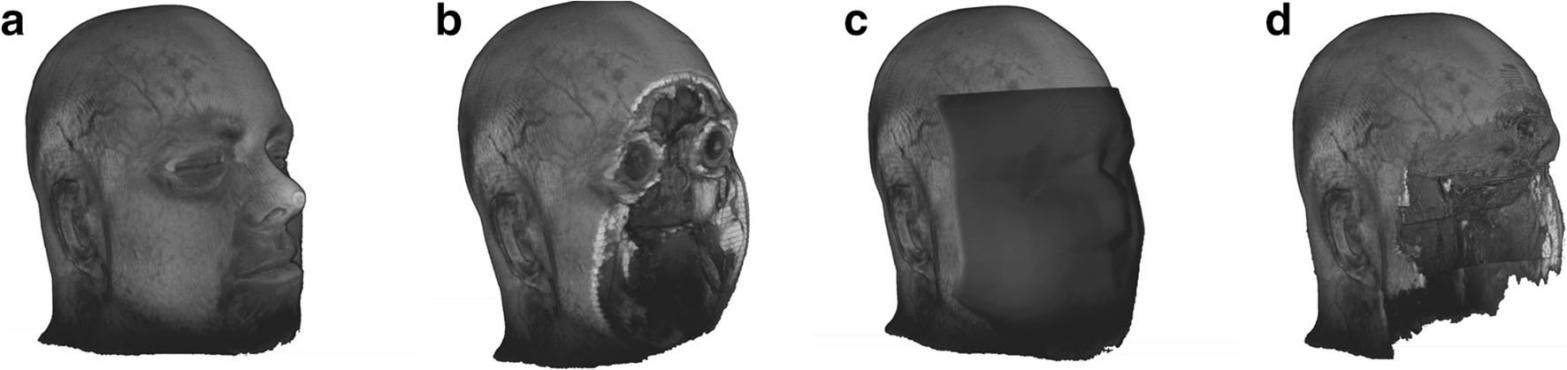
Example 3D-rendered MRI: full (**a**) and after removal of facial features with QuickShear (**b**), FaceMasking (**c**), and Defacing (**d**). The subject gave written informed consent for using data and for displaying rendering in this figure

Three publicly available methods were selected: QuickShear [12], FaceMasking [13], and Defacing [14] (Fig. 1). For all three methods, default settings were used in this study. FaceMasking was applied on all MR modalities separately. QuickShear and Defacing can only remove facial features from 3D T1 images. To remove the facial features from the other images, the full 3D T1 image of each subject was registered to the other full images of the same subject, using FSL-FLIRT (https://fsl.fmrib.ox.ac.uk/fsl/fslwiki/FLIRT [20]), with 12 degrees of freedom before applying the FFR methods. Using the resulting transformation matrices, the 3D T1 image without facial features was transformed to each of the other image spaces, and subsequently binarized and used as a mask to remove the face from the other images.

#### QuickShear

Starting from a user-supplied brain mask, QuickShear [12] uses two algorithms [21, 22] to create a plane that divides the MRI into two parts. One part contains the facial features, and the other part contains the remainder of the head, including the brain. After finding this plane, the intensity of all voxels on the “facial features” side of the plane is set to zero.

In this study, the brain mask was made with FSL-BET (https://fsl.fmrib.ox.ac.uk/fsl/fslwiki/BET) using previously determined optimal settings [23, 24].

#### FaceMasking

FaceMasking [13] deforms the surface of the face with a filter. In this study, the normalized filtering method was used, which is the recommended filter. The method first selects the boundary of the skull and the face, and registers the image volume to an atlas with annotated face coordinates; then, the identified face region of interest layer is normalized and filtered and, finally, transformed back to the original image.

#### Defacing

Defacing [14] uses an algorithm that calculates the probability of voxels being brain tissue or part of the face, based on 10 annotated atlases of healthy controls. Voxels that are labeled as part of the face and have zero probability of being brain tissue are considered to contain facial features, and their signal intensities are set to zero in order to remove the facial features.

### Clinical research outcome measurements

For all three datasets, commonly used, previously validated automated methods were used to obtain clinically relevant outcome measures on the full images (i.e., images without FFR processing) as well as on all images after FFR. In the AD dataset, NBV and unnormalized brain volume (BV) were measured with SIENAX [23]. In the MS dataset, WMLV was measured by segmenting the lesions on the FLAIR images with the lesion prediction algorithm in the lesion segmentation toolbox (LST-LPA) software [25]. In the glioblastoma dataset, the GBV was measured by taking the union of the segmentation of the glioblastoma necrotic core and enhancing tumor generated by BraTumIA [26]. A short description of the methods is provided in the supplementary data.

To provide context to any observed differences between results from full images and images after FFR, reproducibility of SIENAX, LST-LPA, and BraTumIA was assessed. This was done by repeating the analysis on 10 native images per dataset, selected based on the results from the analyses of images after FFR to include in each case 5 images with large effects of FFR and 5 images with small effects of FFR.

### Statistical analyses

First, we investigated whether the FFR methods would successfully process the data, and if the automated methods could successfully process the data after FFR. A method was considered to have failed on a particular input image if the method gave an error or no output. Images were not excluded if the output quality was considered bad by human observers. The percentages of images for which the FFR methods produced output and the percentages of images for which FFR-processed images could be analyzed by the automated methods were calculated.

Next, the impact of the FFR on the outcome measures was evaluated. In order to allow a direct and fair comparison of metrics between FFR methods, only the subjects for whom all three FFR methods produced output and for which both the full images and all FFR-processed images could be analyzed by the subsequent image analysis method were included.

#### Volumetric analyses

The effect of FFR on volumes was evaluated by assessing changes in NBV and BV (AD dataset), WMLV (MS dataset), and GBV (glioblastoma dataset) in three different ways: in data distribution, variability, and systematic differences. First, to assess data distribution, histogram characteristics (median, first and third quartiles, means, and standard deviation) were calculated for four images (1 full; 3 FFR-processed) and difference characteristics (mL and percentage difference) were calculated for 3 FFR-processed images compared with the full image, and scatter plots and Bland-Altman plots were made. Second, to assess variability in the data, whether random we analyzed intra-class correlation coefficient (ICC) for absolute agreement between volumes obtained from full and FFR-processed images [27, 28] with the lower and upper bounds of 95% confidence interval [CI] in parentheses. Third, to assess systematic differences, two-tailed paired *t* tests were performed between volumes measured in full images and those obtained from each of the FFR-processed images, using a Bonferroni-corrected *p* = 0.05 as threshold for statistical significance.

#### Overlap analysis (MS and glioblastoma datasets)

In MS and glioblastoma datasets, we also compared voxelwise differences between the segmentations obtained with and without FFR, because the image analysis methods used in these datasets produce location-sensitive segmentations of the structures of interest. The full dataset is used as “gold standard” and is compared with each of the three FFR-processed datasets separately, quantifying spatial agreement using Dice’s similarity index (SI) [29]:

$$ \mathrm{SI}=\left(2\times \mathrm{TP}\right)/\left(2\times \mathrm{TP}+\mathrm{FP}+\mathrm{FN}\right) $$

where TP, FP, and FN are, respectively, true positive, false positive, and false negative volumes. SI can range from 0 to 1 and SI = 0 means no overlap and SI = 1, a perfect overlap. We calculated the median and first and third quartiles of SI, FP, and FN.

## Results

### Failure of pipelines

**Fig. 2:**
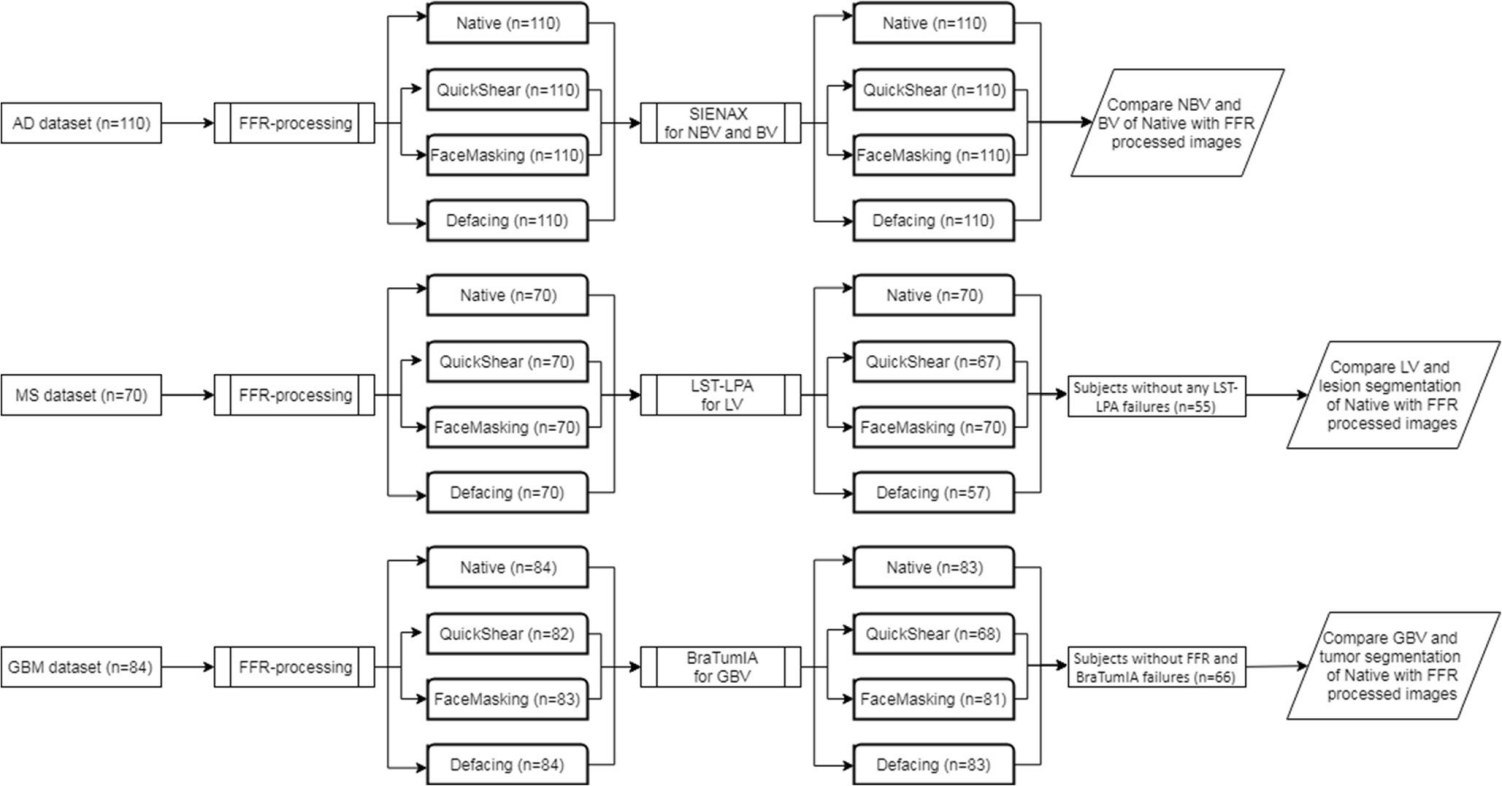
A flowchart summarizing the study steps. Starting with 3 datasets which are FFR-processed, followed with automated (segmentation) methods, selection subjects, and comparing outcome measurements of the FFR-processed images with native images

A simplified flowchart summarizing the study steps is shown in Fig. 2. An overview of the percentages of images for which the FFR methods and automated methods did not fail, i.e., executed without error and with output, is shown for each dataset in Table 2. FFR failed only in the glioblastoma dataset, specifically in 2% of cases for QuickShear and 1% of cases for FaceMasking. Automated method failures varied: while SIENAX completed successfully on all FFR-processed images (AD dataset), LST-LPA produced errors in 4% of cases for QuickShear and 19% of cases for Defacing (MS dataset); and BraTumIA in 17% of cases with QuickShear, 2% with FaceMasking, and 1% with Defacing (glioblastoma dataset). We excluded a subject from further analyses if at least one FFR method failed on this subject. This resulted in 110, 55, and 66 subjects in the AD, MS, and GB datasets, respectively.

**Table 2:** Amount and percentage of images for which FFR methods completed successfully (left half of table) and for which the automated methods SIENAX, LST-LPA, and BraTumIA completed successfully (right half of table)

	Facial features removal			Measurement		
	AD (*n* = 110)	MS (*n* = 70)	Glioblastoma (*n* = 84)	AD (*n* = 110) SIENAX	MS (*n* = 70) LST-LPA	Glioblastoma (*n* = 84) BraTumIA
Full				110/110 100%	70/70 100%	83/84 99%
QuickShear	110/110 100%	70/70 100%	82/84 98%	110/110 100%	67/70 96%	68/82 83%
FaceMasking	110/110 100%	70/70 100%	83/84 99%	110/110 100%	70/70 100%	81/83 98%
Defacing	110/100 100%	70/70 100%	84/84 100%	110/110 100%	57/70 81%	83/84 99%

*AD*, Alzheimer’s disease; *MS*, multiple sclerosis

### Volumetric analysis

#### Full image results

In all datasets, outcome measures obtained from the full images were in the expected range and showed expected distributions. The methods showed good reproducibility, as shown in Table 3, on 10 subjects per dataset, ICC (lower-upper band of 95% CI) of 0.988 (0.973–0.992), 0.998 (0.992–1.000), 0.996 (0.971–0.999), and 0.998 (0.998–1.000) for, respectively, the AD (NBV and BV), MS, and GB datasets. The top rows of Tables 4 and 5 provide the measured values of NBV, BV, WMLV, and GBV for the full images.

**Table 3:** Reproducibility of automated methods on 10 subjects per dataset. From left to right, the table lists median [1st and 3rd quartiles] for volumes; mean ± std for volumes; *p* values for the pairwise comparison of volumes from first and second time processed full images; ICC (absolute agreement (lower-upper band of 95% CI)) between volumes from full and FFR-processed images; and Dice’s similarity index between segmentation from first and second time processed full images

*N* = 10		Volume		*p* value	ICC	SI
AD NBV (L)	First	1.34 [1.29; 1.38]	1.32 ± 0.10			
	Second	1.34 [1.30; 1.40]	1.34 ± 0.07	0.30	0.988 (0.973–0.992)	
AD BV (L)	First	1.09 [1.00; 1.13]	1.06 ± 0.13			
	Second	1.08 [1.30; 1.40]	1.07 ± 0.14	0.35	0.998 (0.992–1.000)	
MS LV (mL)	First	3.00 [1.64; 8.08]	6.39 ± 6.87			
	Second	3.82 [2.11; 8.35]	6.68 ± 6.74	0.16	0.996 (0.971–0.999)	0.95 [0.84–0.98]
GB GBV (mL)	First	39.89 [25.11; 85.92]	68.50 ± 69.66			
	Second	40.65 [25.28; 86.61]	69.38 ± 70.16	0.06	0.998 (0.998–1.000)	0.94 [0.89–0.96]

*ICC*, intra-class correlation coefficient; *N* = amount of subjects; *std*, standard deviation; *AD*, Alzheimer’s disease; *MS*, multiple sclerosis; *SI*, Dice’s similarity index; *FN*, false negative; *FP*, false positive

**Table 4:** Normalized brain volume and brain volume calculated with SIENAX in the AD dataset. From left to right, the table lists median [1st and 3rd quartiles] for volumes; Bonferroni-corrected *p* values for the pairwise comparison of volumes from FFR-processed and full images; volume differences between the full and FFR-processed images as mL and % differences (median [1st and 3rd quartiles]); and ICC (absolute agreement (lower-upper band of 95% CI)) between volumes from full and FFR-processed images

AD *n* = (110)	Normalized brain volume					Brain volume				
	Volume (L)	*p* value	Difference		ICC (lower-upper)	Volume (L)	*p* value	Difference		ICC (lower-upper)
			mL	%				Ml	%	
Full	1.39 [1.34; 1.44]					1.09 [1.01; 1.16]				
QuickShear	1.39 [1.35; 1.44]	0.266	1.26 [− 4.40; 8.62]	0.09 [− 0.32; 0.64]	0.835 (0.767–0.884)	1.09 [1.01; 1.16]	0.001	− 2.44 [− 5.11; − 1.22]	− 025 [− 0.48; − 0.11]	0.982 (0.982–0.993)
FaceMasking	1.40 [1.35; 1.46]	0.003	5.91 [− 1.57; 16.38]	0.44 [− 0.12; 1.19]	0.896 (0.842–0.931)	1.09 [1.01; 1.16]	0.392	− 0.52 [− 2.47; 1.10]	− 0.05 [− 0.2; 0.10]	0.973 (0.960–0.981)
Defacing	1.39 [1.35; 1.44]	1.000	− 2.17 [− 10.80; 7.81]	− 0.16 [− 0.80; 0.55]	0.715 (0.610–0.795)	1.07 [0.99; 1.14]	< 0.001	− 7.60 [− 12.13; − 4.71]	− 0.69 [− 1.14; 0.43]	0.933 (0.8.74–0.961)

Only the images for which FFR was successful and for which the segmentation was successful are included

*ICC*, intra-class correlation coefficient; *n*, number of subjects; *AD*, Alzheimer’s disease

**Table 5:** Lesion volume in the MS dataset and tumor volume in the glioblastoma dataset. From left to right, the table lists median [1st and 3rd quartiles] for volumes; Bonferroni-corrected *p* values for the pairwise comparison of volumes from FFR-processed and full images; volume differences between the full and FFR-processed images as mL and % differences (median [1st and 3rd quartiles]); ICC (absolute agreement (lower-upper band of 95% CI)) between volumes from full and FFR-processed images; Dice’s similarity index between segmentation from full and FFR-processed images; false negative between segmentations from full and FFR-processed images; and false positive between segmentations from full FFR-processed images

	MS; lesion (*n* = 55)							
	Volume (mL)	*p* value	Difference		ICC (lower-upper)	SI	FN (mL)	FP (mL)
			mL	%				
Full	2.71 [1.32; 7.76]							
QuickShear	2.94 [1.35; 8.12]	1.000	0.00 [− 0.08; 0.04]	0.01 [− 2.13; 1.08]	0.992 (0.986–0.995)	0.93 [0.86; 0.95]	0.27 [0.11; 0.76]	0.24 [0.11; 0.74]
FaceMasking	3.15 [1.60; 8.15]	< 0.001	0.23 [− 0.01; 1.26]	4.21 [− 0.64; 25.58]	0.988 (0.972–0.994)	0.90 [0.72; 0.94]	0.24 [0.09; 0.66]	0.58 [0.12; 2.01]
Defacing	2.77 [1.21; 7.77]	0.197	0.00 [− 0.13; 0.02]	− 0.12 [− 3.79; 0.83]	0.998 (0.997–0.999)	0.86 [0.39; 0.94]	0.59 [0.17; 1.35]	0.48 [0.20; 1.10]
	Glioblastoma; tumor (*n* = 66)							
	Volume (mL)	*p* value	Difference		ICC	SI	FN (mL)	FP (mL)
			mL	%				
Full	34.77 [19.54; 53.77]							
QuickShear	29.22 [16.74; 50.06]	< 0.001	− 2.46 [− 7.08; − 0.54]	− 8.11 [− 23.43; − 1.27]	0.843 (0.704–0.912)	0.87 [0.75; 0.92]	5.33 [2.70; 9.74]	1.89 [0.79; 3.80]
FaceMasking	31.65 [14.80; 51.13]	1.000	− 1.31 [− 7.74; 0.57]	− 5.2 [− 16.69; − 2.21]	0.312 (0.074–0.515)	0.86 [0.74; 0.92]	4.81 [2.00; 11.75]	2.19 [1.79; 4.77]
Defacing	28.47 [13.41; 49.21]	< 0.001	− 3.28 [− 8.16; − 0.72]	− 0.70 [− 3.16; − 0.14]	0.810 (0.560–0.901)	0.86 [0.74; 0.92]	5.94 [2.79; 9.75]	1.85 [0.79; 3.21]

Only the images for which FFR was successful and for which the segmentation was successful are included

*ICC*, intra-class correlation; *n*, amount of subjects; *MS*, multiple sclerosis; *SI*, Dice’s similarity index; *FN*, false negative; *FP*, false positive

#### AD dataset

**Fig. 3:**
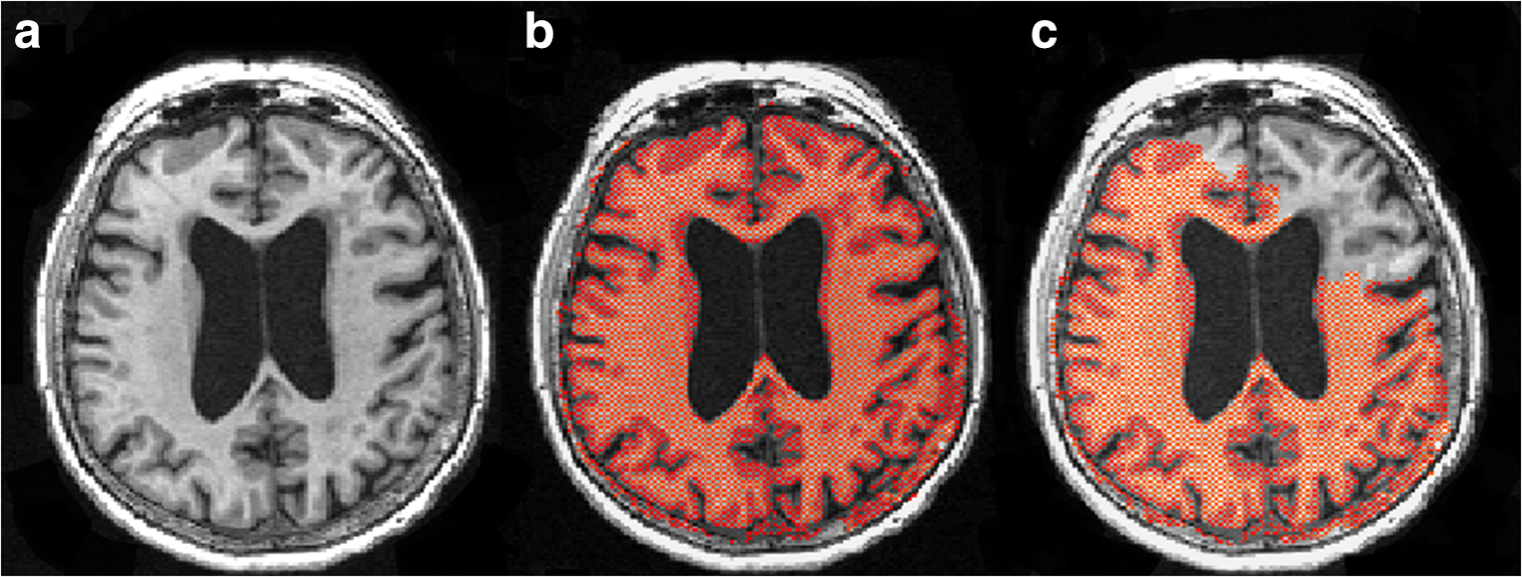
An example of SIENAX affecting the BV by FFR processing showing 3D T1 images (**a**), 3D T1 images with the brain tissue segmentation shown in red (1.04 L) on the full image (**b**), and 3D T1 images with the brain tissue segmentation shown in red (0.87 L) on the FFR-processed image with Defacing (**c**)

**Fig. 4:**
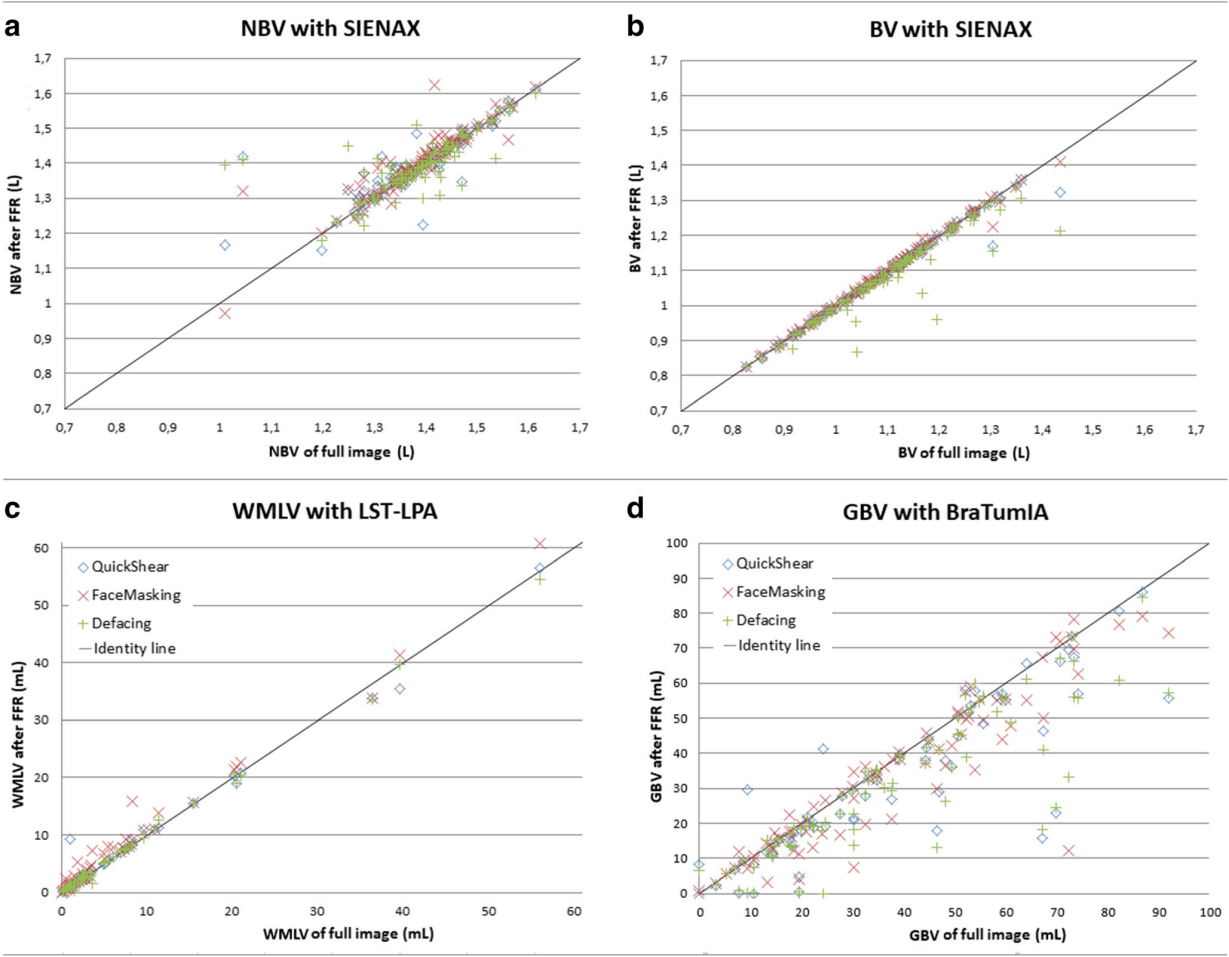
Scatter plots of the normalized brain volume (**a**), brain volume (**b**), white matter lesion volume (**c**), and glioblastoma volume (**d**). The facial removal datasets are plotted against the full scan; QuickShear, blue diamond; FaceMasking, red cross; and Defacing, green plus sign. All scatter plots have an identity line indicating perfect agreement. NBV, normalized brain volume; BV, brain volume; FFR, facial features removal; WMLV, white matter lesion volume; GBV, tumor volume; mL, milliliter; L, liter

Both NBV and BV were affected by FFR processing, in terms of both variability and systematic differences. In Fig. 3, an example of effected SIENAX by FFR processing is given. Figure 4 a and b show scatter plots of NBV and BV for FFR-processed images versus full images in the AD dataset; corresponding Bland-Altman plots are provided in the supplementary section. These results suggest that FFR affected NBV variability more than BV variability, which is confirmed by the ICCs (Table 4): absolute agreement of NBV between FFR-processed images and full images ranged from 0.715 (Defacing) to 0.896 (FaceMasking), while for BV, absolute agreement ranged from 0.933 (Defacing) to 0.982 (QuickShear). Pairwise comparisons showed that NBV was typically overestimated after processing data with QuickShear (median [1st and 3rd quartiles] 1.26 [− 4.40; 8.62] mL) and FaceMasking (5.91 [− 1.57; 16.38] mL), and underestimated after Defacing (− 2.17 [− 10.80; 7.81] mL). BV was typically underestimated after FFR, with median [1st and 3rd quartiles] volume differences of − 2.44 [− 5.11; − 1.22] mL (QuickShear), − 0.52 [− 2.47; 1.10] mL (FaceMasking), and − 7.60 [− 12.13; − 4.71] mL (Defacing). The supplementary section provides means and standard deviations.

#### MS dataset

**Fig. 5:**
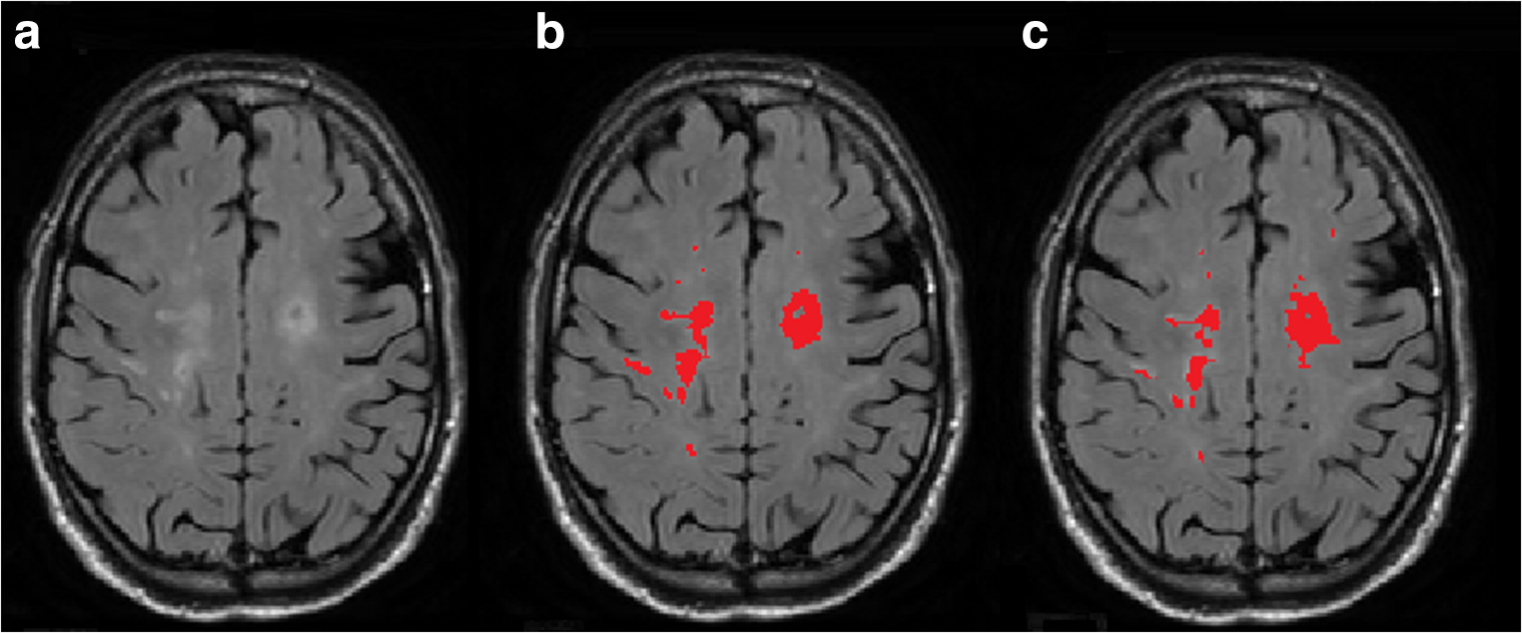
An example of lesion segmentation affected by FFR processing showing 2D FLAIR image (**a**), 2D FLAIR image with the lesion segmentation shown in red (8.30 mL) on full image (**b**), and 2D FLAIR image with the lesion segmentation shown in red (15.91 mL) on FFR-processed image with Defacing (**c**). Dice’s similarity index between the complete 3D segmentations obtained from the full image and FFR-processed image was 0.48

For WMLV, absolute agreement between FFR-processed images and full images was high, but there were small but significant systematic differences. In Fig. 5, an example of affected lesion segmentation by FFR processing is given. Figure 4c shows the WMLV scatter plot; corresponding Bland-Altman plots are provided in the supplementary section. The corresponding ICCs in Table 5 are all ≥ 0.988. The median [1st and 3rd quartiles] WMLV values after FFR were 2.94 [1.35; 8.12] mL (QuickShear), 3.15 [1.60; 8.15] mL (FaceMasking), and 2.77 [1.21; 7.77] mL (Defacing). For the full image, the WMLV value was 2.71 [1.32; 7.76] mL. In the case of FaceMasking, the WMLV is significantly higher than the WMLV of full images, Bonferroni-corrected *p* < 0.001. The supplementary section provides means and standard deviations.

#### Glioblastoma dataset

**Fig. 6:**
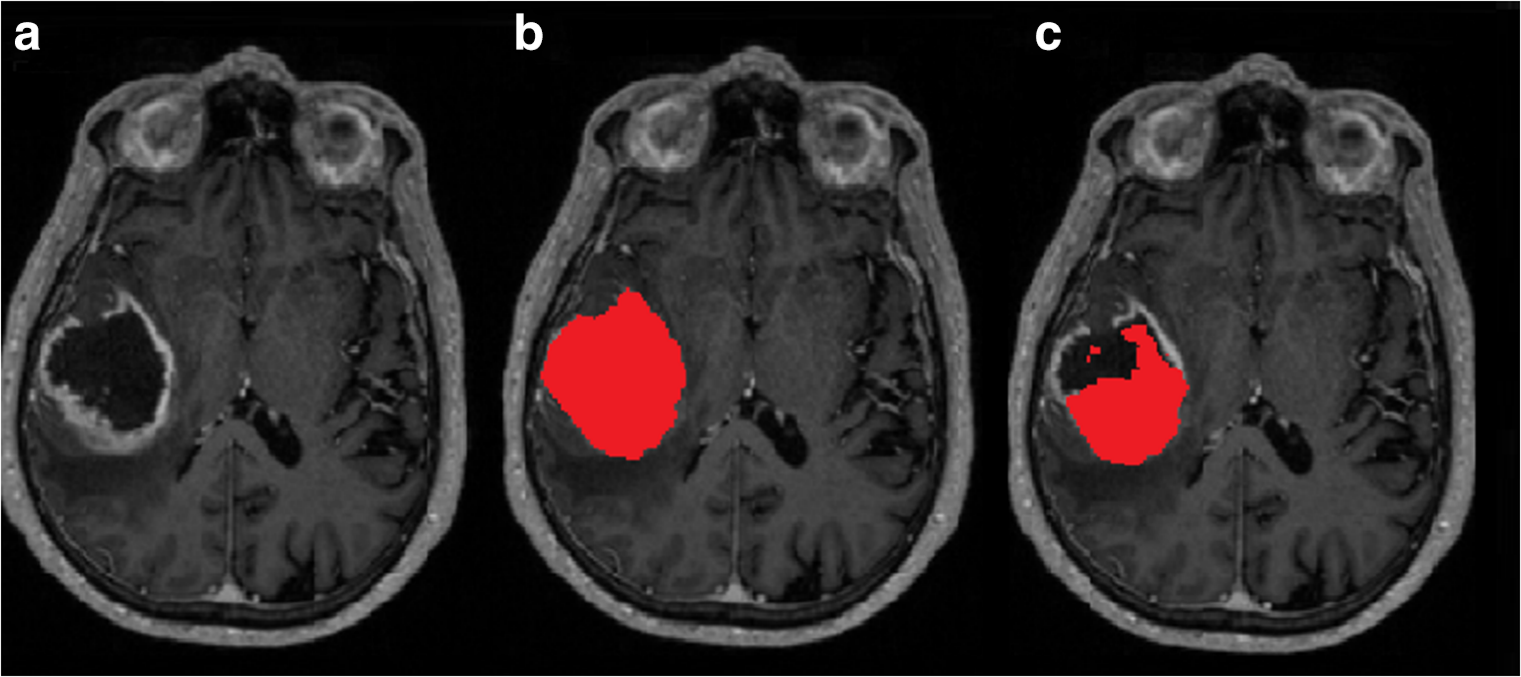
An example of glioblastoma segmentation affected by FFR processing showing 3D T1 post contrast images (**a**), 3D T1 post contrast images with the glioblastoma segmentation shown in red (69.99 mL) on full image (**b**), and 3D T1 post contrast images with the glioblastoma segmentation shown in red (22.87 mL) on FFR-processed image with QuickShear (**c**). Dice’s similarity index between the complete 3D segmentations obtained from the full image and FFR-processed image was 0.48

GBV appeared to be the outcome measure that is most strongly affected by FFR, with low to poor agreement and systematic volume underestimation after FFR by FaceMasking. In Fig. 6, an example of affected glioblastoma segmentation by FFR processing is given. The scatter plot in Fig. 4d, the corresponding Bland-Altman plots in the supplementary section, and the ICCs in Table 5 show that FFR was affected, irrespective of tumor size. ICCs of 0.843, 0.312, and 0.810 between the full and FFR-processed images for QuickShear, FaceMasking, and Defacing indicate substantial effects of FFR. GBV were lower after FFR: differences with full image values were − 2.46 [− 7.08; − 0.54] mL (QuickShear), − 1.31 [− 7.74; 0.57] mL (FaceMasking), and Defacing − 3.28 [− 8.16; − 0.72] mL (Defacing). The supplementary section provides means and standard deviations.

### Overlap analysis

#### MS dataset

Table 5 also presents the spatial agreement of the WM lesion (WML) segmentations. The median [1st and 3rd quartiles] SI values of the WML segmentations from FFR-processed images with the corresponding segmentations from full images were 0.93 [0.86; 0.95] (QuickShear), 0.90 [0.72; 0.94] (FaceMasking), and 0.86 [0.39; 0.94] (Defacing). The FN volumes of FFR-processed images were 0.27 [0.11; 0.76] mL (QuickShear), 0.24 [0.09; 0.66] mL (FaceMasking), and 0.59 [0.17; 1.35] mL (Defacing). The FP volumes were 0.24 [0.11; 0.74] mL (QuickShear), 0.58 [0.12; 2.01] mL (FaceMasking), and 0.48 [0.20; 1.10] mL (Defacing). The supplementary section provides means and standard deviations.

#### Glioblastoma dataset

The SI, FN, and FP of the glioblastoma segmentation are shown in Table 5. The median [1st and 3rd quartiles] SI values of the FFR-processed glioblastoma segmentations with full image segmentations were 0.87 [0.75; 0.92] (QuickShear), 0.86 [0.74; 0.92] (FaceMasking), and 0.86 [0.74; 0.92] (Defacing). The FN volumes were 5.33 [2.70; 9.74] mL (QuickShear), 4.81 [2.00; 11.75] mL (FaceMasking), and 5.94 [2.79; 9.75] mL (Defacing). The FP volumes were 1.89 [0.79; 3.80] mL (QuickShear), 2.91 [1.79; 4.77] mL (FaceMasking), and 1.85 [0.79; 3.21] mL (Defacing). The supplementary section provides means and standard deviations.

## Discussion

When sharing MRI data between research institutions, it is crucial to protect the privacy of participants. In addition to removing identifying metadata from MRI, facial features should also be removed. The current study evaluated how three publicly available FFR methods affect clinically relevant imaging outcome measures in AD, MS, and glioblastoma as derived using commonly used automated methods. Our results showed that the commonly used FFR methods can lead to subsequent failures of automated volumetric pipelines. Moreover, FFR can lead to substantial changes—both random (low ICC) and systematic (significant differences)—in volumes obtained by automated methods. The observed differences in outcome measures between full images and images after FFR cannot be attributed to random variation of SIENAX, LST-LPA, or BraTumIA, because the reproducibility of those methods was high.

The automated methods LST-LPA for WMLV and BraTumIA for GBV failed to successfully execute on multiple FFR-processed images. It should be mentioned that we applied the automated methods with their default settings and did not attempt to remedy the errors. We did, however, assess the failures and we suspect that the failures were related to image registration steps, because registration methods can be susceptible to (disease related) artifacts as recently highlighted by Dadar et al [30]. This recent study showed that registration used in the automated methods could have problems with higher levels of noise and non-uniformity in images and that head size could have an effect on registration methods. It is conceivable that if the face is removed or deformed, the level of noise and non-uniformity could change and lead to failures.

The possible importance of image registration in causing changes after FFR is further suggested by the higher variability of NBV compared with BV after FFR. To compute the NBV, SIENAX multiplies the BV (calculated in native subject space) by a volumetric scaling factor obtained from a linear registration of the brain image to a standard brain image, additionally using a derived skull image. FFR could affect the removal of non-brain tissue and identification of the skull, and thereby cause a different registration result, culminating in altered NBV values. Differences in shapes of the head and face between people (related to, e.g., sex or ethnicity) may affect performance of standard FFR algorithms which may have ramifications especially for subsequent analysis methods that use the skull such as SIENAX. However, there are also cases in which the NBV was not affected by FFR, so maybe there is a cutoff on how much of the head can be removed without affecting the NBV measurement. In a further study, an optimum could be determined between the amount of facial features that should be removed for de-identification and the amount that should remain for correct analyses with automated methods.

Therefore, it would be interesting to study in more detail the effect of FFR methods on registration and other processing steps in a systematic way. Milchenko and Marcus [13] and Bischoff-Grethe et al [14] both addressed the effects of their FFR method on skull stripping; however, it would be interesting to analyze the effects of these methods on other processing steps as well as multiple skull stripping methods, all in the same dataset for an objective comparison. Next, to remedy those errors, analysis methods and processing steps should be made robust against the absence or distortion of facial features. As an example, facial features could be removed from fixed reference images in registration steps or in reference templates (e.g., of tissue probabilities) in image processing pipelines. Moreover, it would also be helpful to study if changing the default settings of the automated methods improves the segmentation.

The measured volumes of the automated methods are affected—both random (low ICC) and systematic (significant differences). The random effects are mostly visible in the volume change of the NBV and GBV after FFR processing and the significant differences are visible in the volume change of BV and GBV. The Bland-Altman plots show that the volume changes are not dependent on the measured volumes. FFR affects not only the measured volumes of the automated methods but also the extent and precise spatial location of the WML and the glioblastomas, as demonstrated by the overlap analyses. For the WML in MS, median FP and FN fractions ranged between about 10 and 25% of the median total WMLV. Similar effects were observed for glioblastoma, with median FN fractions between about 15 and 20%, and median FP fractions between about 6 and 9%. Both the volumetric and spatial results indicate that the differences between full image segmentations and FFR-processed segmentations are substantial. This is unexpected, especially for LV and GBV, as given that both the MS lesions and the glioblastoma are located within the region occupied by brain tissue that should not be, and judging from our visual inspections indeed was not, affected by the FFR methods. Both in MS and glioblastoma, the exact location and extent of pathological changes are of importance; therefore, these artifactual post FFR segmentation changes should be investigated in more detail and methods should be devised and tested to mitigate these effects.

Our results showed that the effects of FFR on current methods are a common problem across domains: brain volumes, MS lesion volumes, and glioblastoma volumes were all to some degree affected by FFR. The next step would be to investigate how to overcome such issues for SIENAX, LST-LPA, and BraTumIA, or in a broader sense, to study and mitigate sources of error after FFR for multiple methods aimed at brain volume, MS lesion, or glioblastoma segmentation. Another option would be to consider removing the facial features from fixed reference images in registration steps or in reference templates (e.g., of tissue probabilities) in image processing pipelines.

It should be noted that in this study, we did not test if the FFR methods indeed made the participant unrecognizable. However, we observed that the FFR methods in some cases seemed to leave parts of the face intact. We did not assess whether this made the person recognizable, because this would require a more rigorous setup outside the scope of this study. However, it would be important to establish guidelines on how to make participants unrecognizable, specifically which parts of the face should be removed or otherwise processed to ensure participants’ privacy. Moreover, for protecting participants’ privacy, it may be important to take into account that reconstruction of removed or deformed facial features may be possible [31].

In conclusion, this study highlighted a new challenge to the neuroimaging research community, which is to ensure high-quality neuroimaging research while protecting participants’ privacy. Our results demonstrate that facial features removal of brain MRI can lead both to failure of automated analysis methods (mostly by LST-LPA and BraTumIA) and to changes in volumes obtained by the analysis methods, including both “random” variation (mostly by NBV and GBV) and systematic differences (mostly by BV and GBV). Therefore, volumetric image analysis methods need to be carefully assessed and optimized with regard to FFR methods, in order to ensure the reliability of clinical research outcomes while protecting participants’ privacy in multicentric, collaborative studies. This could be done by improving image registration accuracy after FFR, addressing in more detail the effect of FFR methods on other processing steps or by developing methods that are tailored to images from which facial features have been removed.

## Electronic supplementary material

ESM 1 (DOCX 13339 kb)

ESM 2 (DOCX 20.4 kb)

## Abbreviations

AD: Alzheimer’s disease
BV: Unnormalized brain volume
EDSS: Expanded Disability Status Scale
FFR: Facial features removal
FLAIR: Fluid-attenuated inversion recovery
FN: False negative
FP: False positive
GBV: Tumor volume of glioblastoma patients
ICC: Intra-class correlation coefficient
LST-LPA: Lesion prediction algorithm in lesion segmentation toolbox
MCI: Mild cognitive impairment
MPRAGE: Magnetization-prepared rapid acquisition gradient echo
MR: Magnetic resonance
MRI: Magnetic resonance imaging
MS: Multiple sclerosis
NBV: Normalized brain volume
SI: Dice’s similarity index
T: Tesla
WML: White matter lesion
WMLV: White matter lesion volume
