# Supplementary material for: Facing privacy in neuroimaging: removing facial features degrades performance of image analysis methods
Source: Eur Radiol. 2019 Nov 5;30(2):1062–74. doi: 10.1007/s00330-019-06459-3 (PMC6957560; doi:10.1007/s00330-019-06459-3)
Supplement: Supplementary file 1 — (DOCX 13339 kb) [file 330_2019_6459_MOESM1_ESM.docx]

**
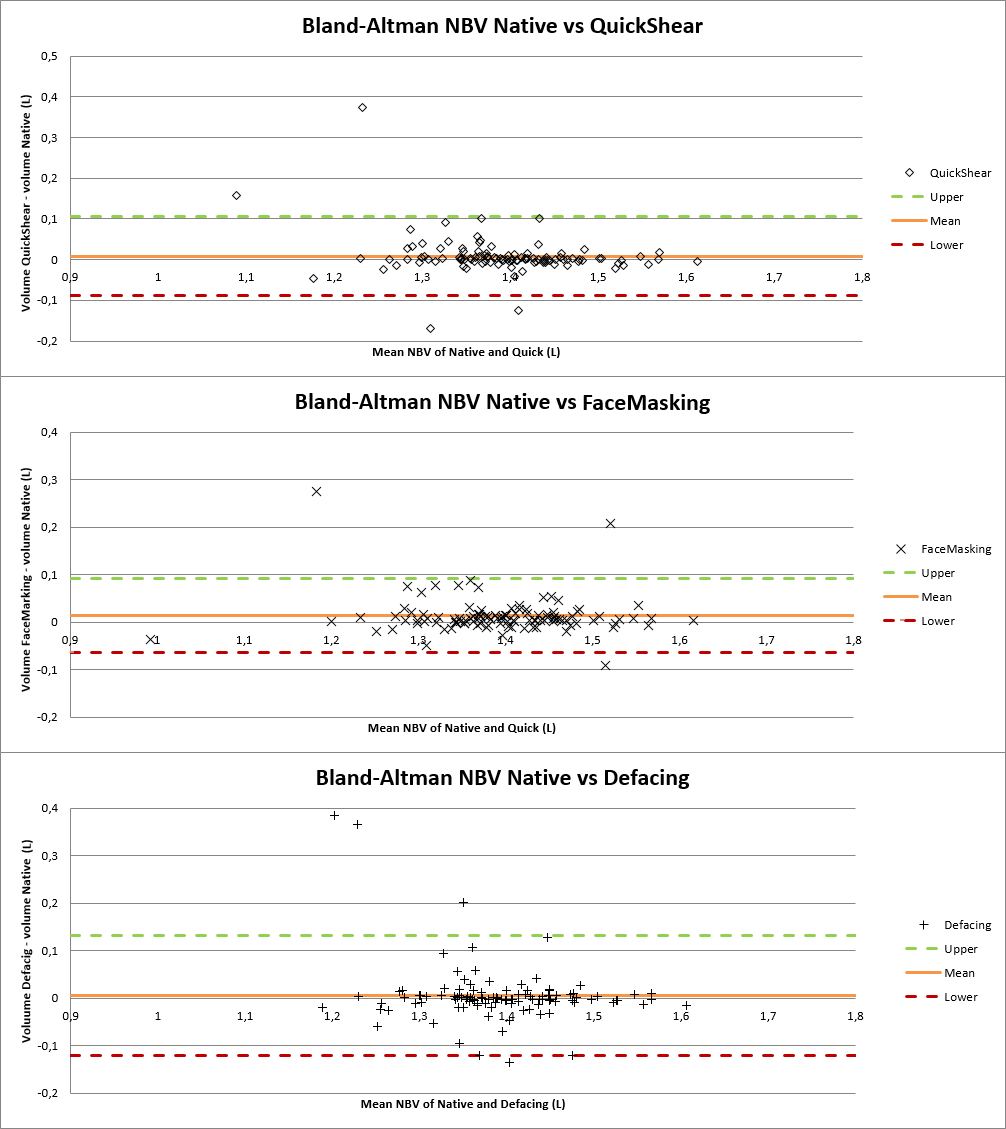
**

**Supplementary Figure 1: Bland-Altman plot of the normalized brain volume for QuickShear (diamond), FaceMasking (cross) and Defacing (plus sign). Bland-Altman plot are between the normalized brain volume of the Native images and FFR-processed images. Normal line = mean of volume FFR-processed images – volume Native image, dotted line = upper and lower bound are of 95% confidence interval.
 Abbreviations: NBV = normalized brain volume, FFR = facial features removal and L = liter.**

**
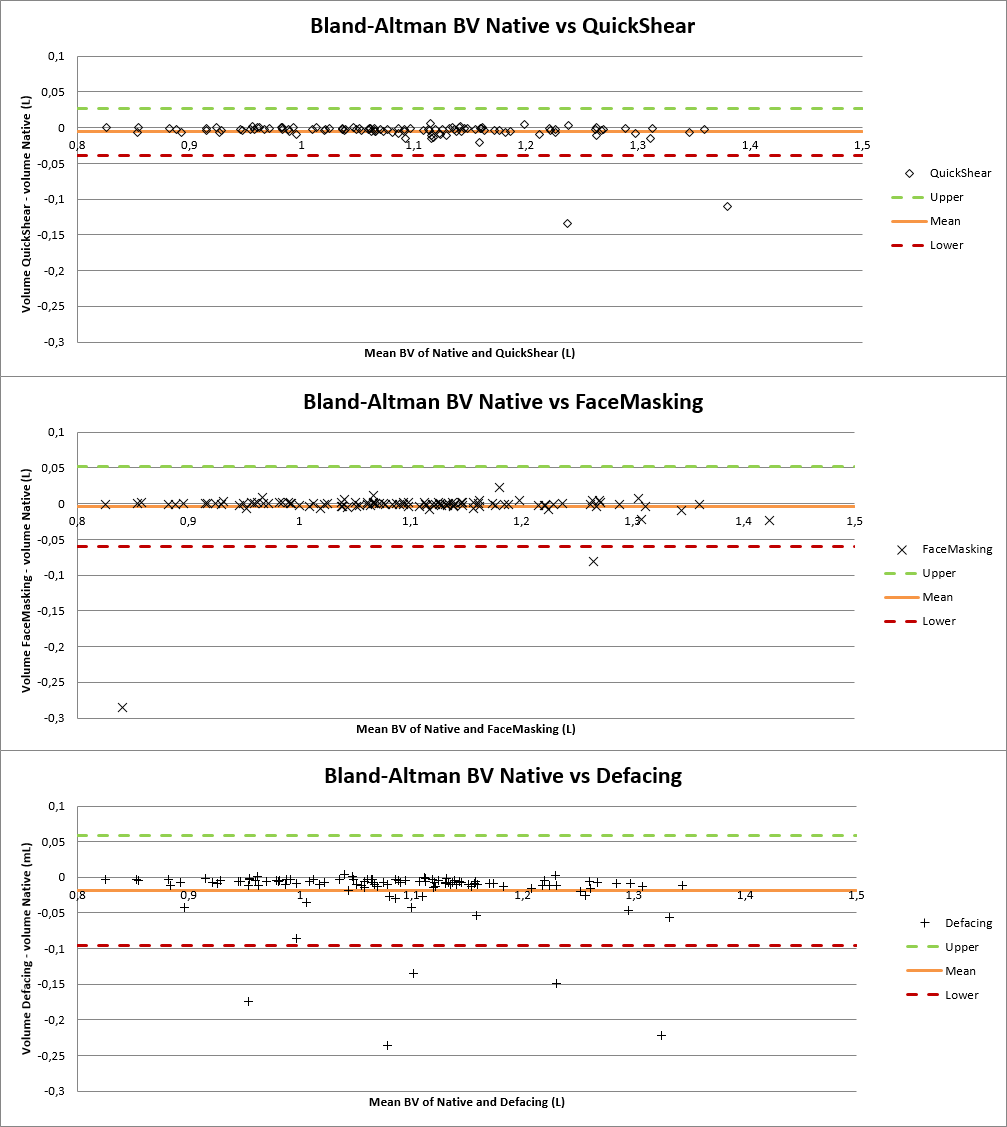
**

**Supplementary Figure 2: Bland-Altman plot of the brain volume for QuickShear (diamond), FaceMasking (cross) and Defacing (plus sign). Bland-Altman plot are between the brain volume of the Native images and FFR-processed images. Normal line = mean of volume FFR-processed images – volume Native image, dotted line = upper and lower bound are of 95% confidence interval.
 Abbreviations: BV = brain volume, FFR = facial features removal and L = liter.**

**
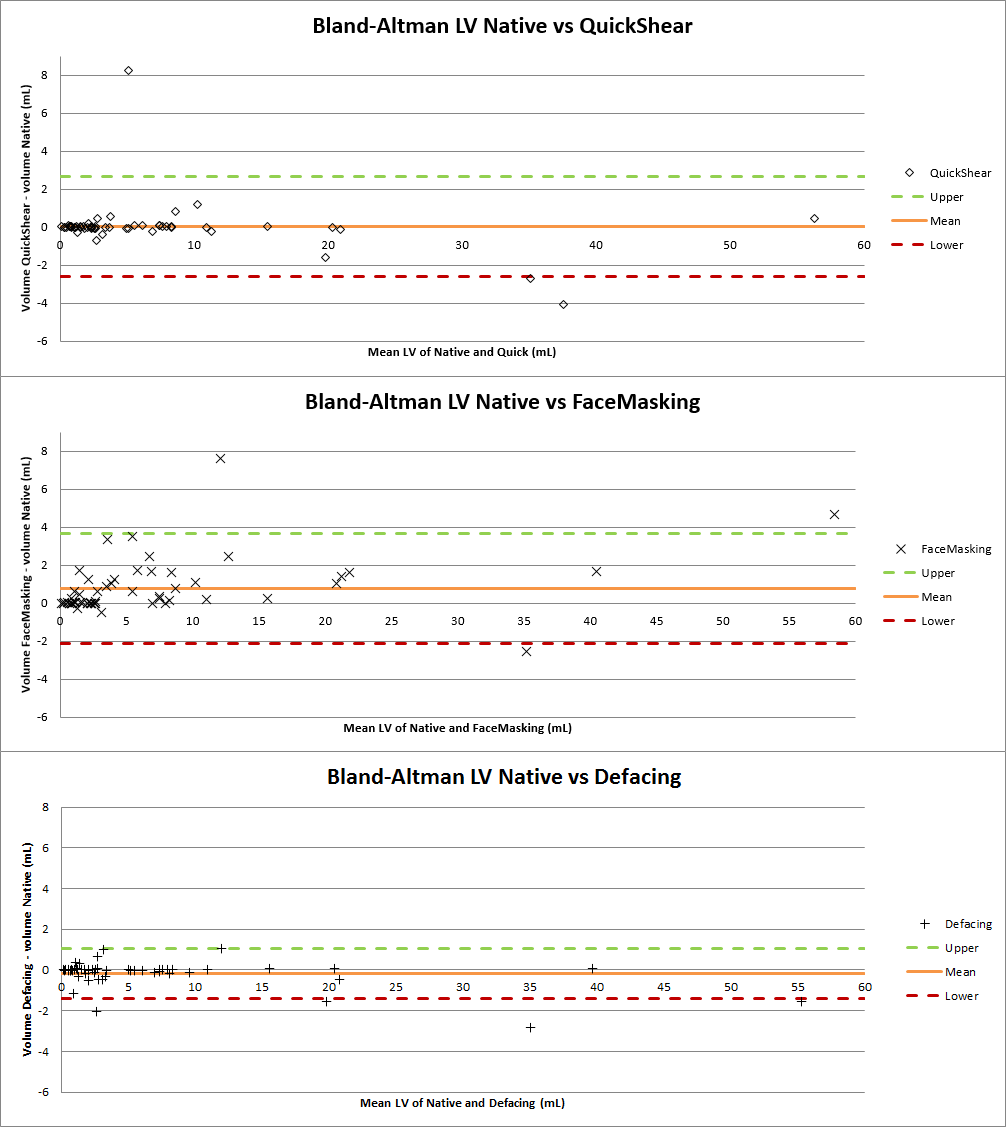
**

**Supplementary Figure 3: Bland-Altman plot of lesion volume for QuickShear (diamond), FaceMasking (cross) and Defacing (plus sign). Bland-Altman plot are between the lesion volume of the Native images and FFR-processed images. Normal line = mean of volume FFR-processed images – volume Native image, dotted line = upper and lower bound are of 95% confidence interval.
 Abbreviations: LV = lesion volume, FFR = facial features removal and mL = milliliter.**

**
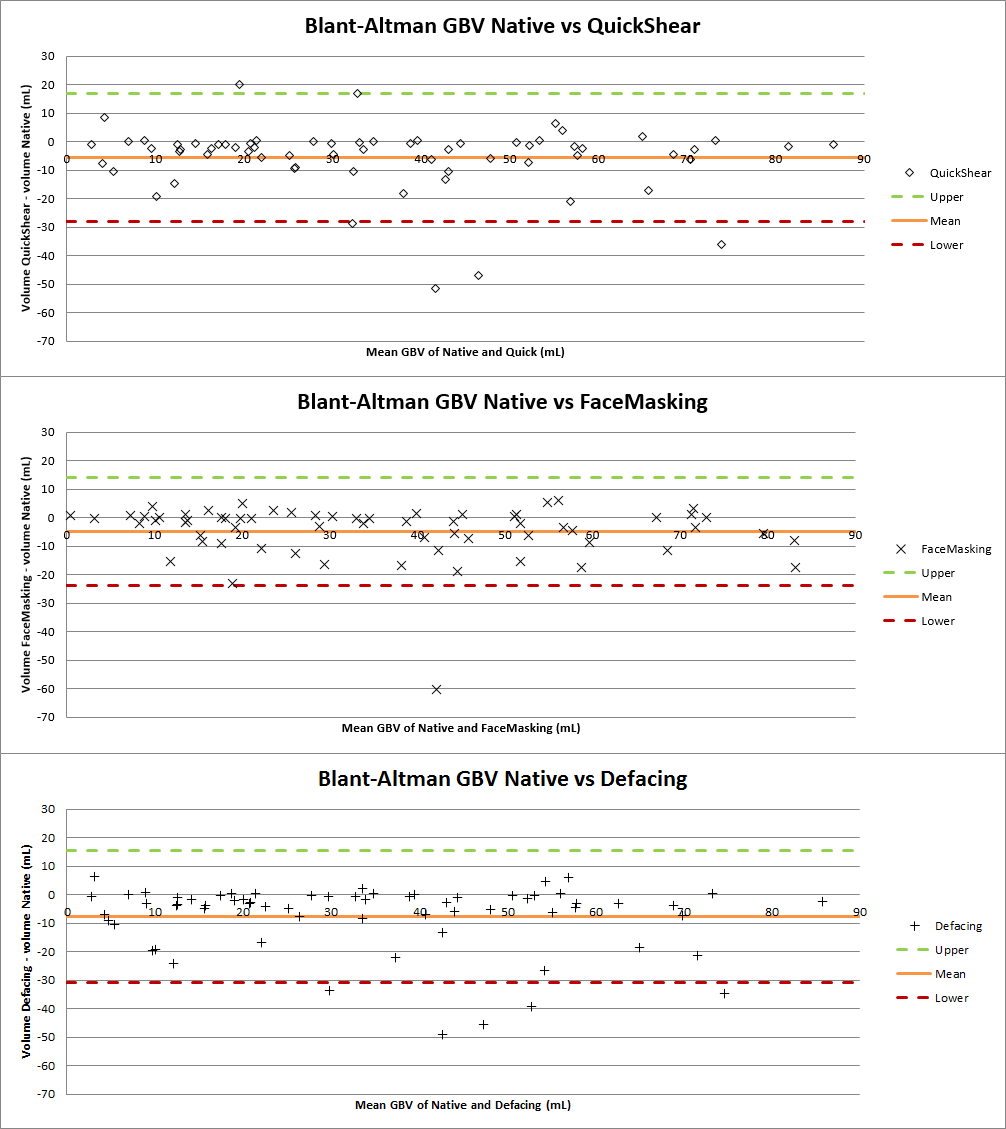
**

**Supplementary Figure 4: Bland-Altman plot of glioblastoma volume for QuickShear (diamond), FaceMasking (cross) and Defacing (plus sign). Bland-Altman plot are between the glioblastoma volume of the Native images and FFR-processed images. Normal line = mean of volume FFR-processed images – volume Native image, dotted line = upper and lower bound are of 95% confidence interval.
 Abbreviations: GBV = glioblastoma volume, FFR = facial features removal and mL = milliliter.**
