# Supplementary material for: Facing privacy in neuroimaging: removing facial features degrades performance of image analysis methods
Source: Eur Radiol. 2019 Nov 5;30(2):1062–74. doi: 10.1007/s00330-019-06459-3 (PMC6957560; doi:10.1007/s00330-019-06459-3)
Supplement: Supplementary file 2 — (DOCX 20.4 kb) [file 330_2019_6459_MOESM2_ESM.docx]

**Supplementary Tables**

| **Dataset** | **n (% females)** | **Age in years** | **Disease type** | **DD** | **EDSS** |
| --- | --- | --- | --- | --- | --- |
| AD | 110 (23%) | 75 (60-87) | HC=39, MC =52, AD=19 |  |  |
| MS | 70 (67%) | 34.9 (17-52) |  | 7.6 (1.0-28.0) | 2.0 (0.0-6.5) |
| GB | 84 (38%) | 61.4 (21-84) |  |  |  |
| ***Supplementary Table 1: Demographics of AD, MS and GB datasets. From left to right, the Table lists n (% female), age [mean (range)], disease type AD dataset, disease duration of MS dataset, EDSS [median (range)] of MS dataset.***  ***Abbreviations: n = number of subjects, AD = Alzheimer’s Disease, MS = multiple sclerosis, HC = healthy elderly control, MCI = Mild cognitive impairement, DD = disease duration, EDSS = Expanded Disability Status Scale*** | | | | | |

| **AD n = (110)** | **Normalized Brain Volume** | | | | **Brain Volume** | | | |
| --- | --- | --- | --- | --- | --- | --- | --- | --- |
|  | Volume (L) | p-value | Difference (mL)/(%) | ICC | Volume (L) | p-value | Difference (mL) | ICC |
| Full | 1.39±0.09 |  |  |  | 1.09±0.12 |  |  |  |
| QuickShear | 1.40±0.08 | 0.266 | 8.07±49.26  0.73±4.32 | 0.835 | 1.09±0.12 | 0.001 | -5.72±16.56  -0.48±1.23 | 0.982 |
| FaceMasking | 1.40±0.09 | 0.003 | 12.83±39.64  0.99±3.30 | 0.896 | 1.09±0.12 | 0.392 | -4.13±-28.45  -0.38±2.84 | 0.973 |
| Defacing | 1.39±0.08 | 1.000 | 5.51±64.400  0.61±5.73 | 0.715 | 1.08±0.11 | <0.001 | -18.60±39.17  -1.61±3.22 | 0.933 |
| ***Supplementary Table 2: Normalized Brain Volume and Brain Volume calculated with SIENAX in the AD-dataset. From left to right, the Table lists*** ***mean±std for volumes; Bonferroni-corrected p-values for the pairwise comparison of volumes from FFR-processed and full images; volume differences between the full and FFR -processed images as mL differences (mean±std), and ICC (absolute agreement) between volumes from full and FFR-processed images.***  ***Only the images for which FFR was successful and for which the segmentation was successful are included.***  ***Abbreviations: ICC = intra-class correlation coefficient, std = standard deviation, n = number of subjects, AD = Alzheimer’s Disease.*** | | | | | | | | |

|  | **MS; Lesion (n = 55)** | | | | | | | **Gioblastoma; Tumor (n = 66)** | | | | | | |  |  |
| --- | --- | --- | --- | --- | --- | --- | --- | --- | --- | --- | --- | --- | --- | --- | --- | --- |
|  | Volume (mL) | p-value | Difference (mL)/(%) | ICC | SI | FN (mL) | FP (mL) | Volume (mL) | p-value | Difference (mL)/(%) | ICC | SI | FN (mL) | FP (mL) | | |
| Full | 6.95±10.53 |  |  |  |  |  |  | 38.56±23.09 |  |  |  |  |  |  |  |  |
| QuickShear | 6.98±10.15 | 1.000 | 0.03±1.35  15±113 | 0.992 | 0.76±0.35 | 1.52±5.35 | 1.55±4.93 | 33.03±21.48 | <0.001 | -5.52±11.51  1323±10865 | 0.843 | 0.74±0.30 | 9.80±12.41 | 4.28±8.25 | | |
| FaceMasking | 7.73±11.01 | <0.001 | 0.78±1.47  22±51 | 0.988 | 0.77±0.28 | 0.84±1.85 | 1.62±25.07 | 27.72±44.38 | 1.000 | 0.16±41.61  130±990 | 0.312 | 0.76±0.26 | 8.64±10.84 | 8.86±39.86 | | |
| Defacing | 6.79±10.25 | 0.197 | -0.16±0.62  -2±17 | 0.998 | 0.66±0.38 | 1.93±5.43 | 1.77±5.41 | 30.90±11.51 | <0.001 | -7.66±11.88  -4.05±8.12 | 0.810 | 0.74±0.30 | 10.25±11.80 | 2.53±2.74 | | |
| ***Supplementary Table 3: Lesion volume in the MS-dataset and tumor volume in Glioblastoma-dataset. From left to right, the Table lists mean±std for volumes; Bonferroni-corrected p-values for the pairwise comparison of volumes from FFR-processed and full images; volume differences between the full and FFR -processed images as mL differences (mean±std), ICC (absolute agreement) between volumes from full and FFR-processed images, dice similarity index between segmentation from full and FFR-processed images, false negative between segmentations from full and FFR-processed images and false positive between segmentations from full FFR-processed images. .***  ***Only the images for which FFR was successful and for which the segmentation was successful are included.***  ***Abbreviations: ICC = intraclass correlation, n = amount of subjects, std = standard deviation, MS = multiple sclerosis, SI = dice similarity index, FN = false negative and FP = false positive.*** | | | | | | | | | | | | | | | |  |
